# Inoculation for Syphilis

**Published:** 1853-03

**Authors:** 


*Inoculation for Syphilis.* — A plan has lately been proposed by M. Auzias Turenne, of Paris, to inoculate with the syphilitic virus till the system becomes proof against the disease! Messrs. Malgaigne and Depaul, Parisian surgeons of prominence, have advocated the plan. We know not what evidence has been advanced by these gentlemen of the efficacy of this method of protection; but we think they should at the same time suggest a safeguard against the moral taint of licentiousness! The proposition shows that the subject has, by them, been regarded as one of importance only in its physical relations. It is stated that the subject excited an animated discussion before the Academy of Medicine, of Paris, and that, to the credit of the Academy, the practice was repudiated by all the members excepting the gentlemen named.

